# A new ketogenic formulation improves functional outcome and reduces tissue loss following traumatic brain injury in adult mice

**DOI:** 10.7150/thno.48995

**Published:** 2021-01-01

**Authors:** Orli Thau-Zuchman, Linda Svendsen, Simon C. Dyall, Ursula Paredes-Esquivel, Molly Rhodes, John V. Priestley, René G. Feichtinger, Barbara Kofler, Susanne Lotstra, J. Martin Verkuyl, Robert J. Hageman, Laus M. Broersen, Nick van Wijk, Jose P. Silva, Jordi L. Tremoleda, Adina T. Michael-Titus

**Affiliations:** 1 Centre for Neuroscience, Surgery and Trauma, The Blizard Institute, Barts and The London School of Medicine and Dentistry, Queen Mary University of London, 4 Newark Street, Whitechapel, London, E1 2AT, UK.; 2 University of Roehampton, Department of Life Sciences, Grove House, Roehampton Lane, London, SW15 5PJ, UK.; 3 Research Program for Receptor Biochemistry and Tumor Metabolism, Department of Pediatrics, University Hospital of the Paracelsus Medical University, Müllner Hauptstrasse 48, A-5020, Salzburg, Austria.; 4 Danone Nutricia Research, Uppsalalaan 12, Utrecht, 3584 CT, The Netherlands.

**Keywords:** Adult traumatic brain injury, epigenetic modifications, ketogenic diet, neurological outcome, neuroprotection

## Abstract

**Rationale:** Traumatic brain injury (TBI) leads to neurological impairment, with no satisfactory treatments available. Classical ketogenic diets (KD), which reduce reliance on carbohydrates and provide ketones as fuel, have neuroprotective potential, but their high fat content reduces compliance, and experimental evidence suggests they protect juvenile brain against TBI, but not adult brain, which would strongly limit their applicability in TBI.

**Methods:** We designed a new-KD with a fat to carbohydrate plus protein ratio of 2:1, containing medium chain triglycerides (MCT), docosahexaenoic acid (DHA), low glycaemic index carbohydrates, fibres and the ketogenic amino acid leucine, and evaluated its neuroprotective potential in adult TBI. Adult male C57BL6 mice were injured by controlled cortical impact (CCI) and assessed for 70 days, during which they received a control diet or the new-KD.

**Results:** The new-KD, that markedly increased plasma Beta-hydroxybutyrate (β-HB), significantly attenuated sensorimotor deficits and corrected spatial memory deficit. The lesion size, perilesional inflammation and oxidation were markedly reduced. Oligodendrocyte loss appeared to be significantly reduced. TBI activated the mTOR pathway and the new-KD enhanced this increase and increased histone acetylation and methylation.

**Conclusion:** The behavioural improvement and tissue protection provide proof of principle that this new formulation has therapeutic potential in adult TBI.

## Introduction

Traumatic brain injury (TBI) is a leading cause of death and disability worldwide 1. The primary injury leads to tissue disruption, loss of neuronal and non-neuronal cells, neuroinflammation, oxidative stress, and brain dysconnectivity 2. These changes underlie the functional sensorimotor and cognitive deficits seen post-trauma. At present there is no satisfactory treatment and furthermore, TBI increases the risk of neurodegenerative diseases, such as dementia 3 and is also associated with the risk of developing post-traumatic epilepsy 4.

In parallel with brain structure disruption, TBI leads to altered cerebral energy metabolism. Under normal conditions, glucose is the main energy-producing source in the brain. TBI leads to an acute glucose hypermetabolism 5, followed by a prolonged cerebral glucose hypometabolism. Under these conditions, the brain can switch to the use of an alternative fuel, such as ketone bodies (KB) 5. KB, i.e. ß-hydroxybutyrate, acetoacetate and acetone, are synthesized from fatty acids in the liver. KBs can be produced under conditions of caloric restriction or during use of ketogenic diets (KDs). KDs are characterised by a high fat and low carbohydrate content, and their use leads to the development of a state of ketosis, i.e. increase in plasma and tissue KB levels 6. The KD dietary concept was first developed in the 1920s as a therapy for epilepsy and has been in clinical use worldwide as a non-pharmacological treatment option for treatment-resistant epilepsy 7. KD-based interventions are well- established in pediatric epilepsy 8 but less so in adult epilepsy 9. They have significant efficacy in reducing seizures, but these fat-rich diets have low palatability and can lead to adverse effects, such as tiredness, excessive thirst, kidney stones and constipation, which significantly reduce patient compliance 10.

Apart from epilepsy, KD interventions have been shown to have neuroprotective potential in a variety of other neurological indications 9, 11, 12. However, in TBI the experimental evidence generated in rodents so far with classical KD (with ratios of fat to protein and carbohydrate of 4:1 or higher), suggests that the neuroprotective effect of such an intervention would be limited to the protection of the juvenile brain only 13-15.

In the present study, we tested a new-KD formulation in an experimental model of TBI, the controlled cortical impact (CCI) model, in adult mice. The new-KD formulation contained medium-chain triglycerides (MCT) and also the long-chain polyunsaturated fatty acid docosahexaenoic acid (DHA), which has been shown to have intrinsic neuroprotective properties in TBI 16, 17. It also contained low glycaemic index carbohydrates, fibres, and the ketogenic amino acid leucine, with a resulting overall ratio of fat to protein and carbohydrate of 2:1. We hypothesised that this composition would increase the neuroprotective effect of the diet, while maintaining its ketogenic potential and also increasing its palatability and reducing the adverse effects of the classical KD for more compliant use in patients. As the energy demise after TBI occurs very rapidly, we fed the animals this diet immediately after injury and maintained them on it for 70 days, while monitoring their recovery. We show that this new-KD formulation improves several aspects of neurological outcome and significantly counters the tissue damage and the increased inflammation and oxidation triggered by injury, and thus we present the first experimental evidence that a KD designed this way could confer neuroprotection in adult TBI.

## Materials and Methods

### Animals

A total of 30 adult, 10-12 week-old male C57BL/6 mice, weighing 22-27 g (Charles River Laboratories, Margate, UK), were housed in groups of five, maintained the same throughout the study, in Individually Ventilated Cage (IVC; Allentown Europe, UK), in a 12 h light-dark cycle, with controlled room temperature (21 ± 1 °C) and relative humidity (40-60%) and with diet and water ad libitum. Health screens provided by the vendor indicated that animals were free of known pathogens in accordance with FELASA guidelines. A 7-day acclimatization phase occurred prior to the study. Food intake and body weight were monitored daily. All animal procedures were carried out under a Project Licence approved by the Animal Welfare and Ethical Review Body, at Queen Mary University of London and the UK Home Office, in accordance with the EU Directive 2010/63/EUT.

### Controlled cortical impact model

A controlled cortical impact (CCI) TBI model was used, as previously 18. After 1-week acclimatisation, mice were anaesthetized using ketamine (50 mg/kg) and medetomidine (0.5 mg/kg; i.p.). Mice were placed in a stereotaxic frame and a midline longitudinal incision was performed to expose the skull. A right lateral craniotomy was carried out using a pneumatic drill, 2.0 mm behind bregma and 2.5 mm lateral to the midline. CCI injury was induced using the following settings: a 3 mm impactor tip with a speed of 3 m/s, a depth of 2.2 mm and a dwell time of 100 ms, applied using the PCI3000 Precision Cortical Impactor™ (Hatteras Instruments, Inc., US). A control group underwent craniotomy only. After injury, the skull flap was placed back, to allow for expansion, and the skin was sutured. Mice were allowed to recover in an incubator (37°C) until fully awake and active. Buprenorphine (0.05 mg/kg, s.c.) was used in all animals pre-operatively for pre-emptive analgesia and post-operatively every 12 h for 3 days post-surgery.

### Dietary supplementation

Following CCI, mice were randomized into two groups and fed with a control diet ('CCI- Control'; n=10) or with the new preparation of a ketogenic diet (KD) ('CCI-KD'; n=10) for 70 days (detailed composition in Table 1). The craniotomy group were fed with control diet (craniotomy; n=10). The diets were formulated by Danone Nutricia Research (Utrecht, The Netherlands) and manufactured by Ssniff (Soest, Germany). The caloric value of the control and new diet were 377 kcal/100g and 626 kcal/100g, respectively. The comparison between this diet and various other diets with variable ratios fat: carbohydrate and protein is shown in Table S1. Diets were stored at -20 °C to prevent lipid peroxidation, and fresh diet was given daily. No significant differences were seen in the body weight (Figure S1A) and food intake curves between the various experimental groups, throughout the study. All the behavioural work (the primary study endpoint) and tissue analysis, was assessed in “blind”, with the researcher unaware of the treatment, in accordance with the ARRIVE guidelines 19.

### Experimental design and Behavioural testing

The testing carried out on various days post-injury (dpi) is summarised in Figure S1B. Sample size was estimated using power analysis (https://eda.nc3rs.org.uk/eda/) for pairwise post-hoc comparisons after ANOVA, at a statistical power of 90%, with a significance level α < 0.05.

### Modified Neurological Severity Score (mNSS)

The mNSS was used as previously 20, 21, using a scoring based on the ability to perform ten tasks that evaluate motor ability, balance and alertness (Figure S2A). For each performance criterion, the animal can receive a score of 0 (no deficit) or 1 (deficit observed), on occasions 0-3 for increasing severity of deficit. The first mNSS was obtained 24 h after TBI. During the first week, testing was performed every other day, then once a week, until the end of the experiment.

### Rotarod

The Rotarod test (Ugo Basile, France; 3 cm diameter) was used for the evaluation of motor coordination and balance. Prior to surgery, mice were trained on the Rotarod for three consecutive days. The first two trials were 60 s each, at 3 rpm, followed by a single trial at accelerating speed (3 to 20 rpm over 300 s, with intervals of at least 25 min rest). The rest of the training days consisted of one trial at 3 rpm followed by two accelerating trials. The latency to fall from the Rotarod was recorded. The average of all accelerating phase scores was considered as the baseline (pre-injury score). Mice were tested during day 1-3 post- injury, in three trials a day, using the accelerating mode.

### Morris Water Maze

The Morris water maze test (MWM) was used to assess memory associated with spatial learning between days 13-18 post-CCI. A 100 cm diameter pool filled with opaque water at 23 °C was placed inside a white tent, ensuring light uniformity, with 4 visible cues hung 10 cm from the pool walls and a 11 cm diameter Plexiglas resting platform submerged 0.5 cm below water level. Swimming performance (e.g. path, distance, speed and latency) was tracked using software (ANYmaze, Smart, Bioseb, France). A learning period of 5 consecutive days (days 13-17 post-injury) and a probe trial on day 6 (day 18 post-injury) were used. During the learning period, each mouse was subjected to 4 trials a day, in the pool divided into 4 virtual quadrants. The position of the platform was constant throughout the training session, while the starting position on each of the 4 training trials was changed. If a mouse did not find the platform within 60 s, it was guided to it. After reaching the platform, mice were allowed to stay there for 15 s. During the probe trial, mice were allowed to swim for 60 s in the absence of the platform, and the time it took to first enter the quadrant that had previously hosted the platform, was measured.

### BrdU injections

From day 63 post-injury or craniotomy, and for 7 sequential days, animals received i.p. injections of 5-bromo-2-deoxyuridine (BrdU; 50 mg/kg, twice a day), to assess cell proliferation.

### Histology and immunohistochemistry

At day 70 post-TBI, 5 animals from each group were deeply anaesthetized with sodium pentobarbital (50 mg/kg, i.p.; Sagatal, Rhone Merieux, Harlow, UK), and received a transcardiac perfusion with phosphate-buffered saline (PBS; 0.01 M, pH 7.4), followed by 4% paraformaldehyde (PFA) in phosphate buffer (0.1 M, pH 7.4, 4 °C). The brains were dissected out, fixed and paraffin-blocked for histology and immunohistochemistry (IHC) analyses. 7 μm sections were deparaffinised and hydrated (xylene and ethanol). Sections were subjected to antigen retrieval (10 mM citrate buffer, pH 6.0, 30 min at 80 °C), then cooled at room temperature. The tissue was blocked with 5% normal donkey serum in 0.2% Triton X-100 in PBS for an hour, followed by three PBS washes. The primary antibodies used were (overnight incubation): rat anti-BrdU (1:200; Acris Antibodies GmbH, Cat# SM1667PS), rabbit anti- glial fibrillary acidic protein (GFAP) (1:800; Dako, Cat# Z0334), goat anti-Iba-1(1:800; Wako, Cat# 019-19741) and rabbit anti-translocator protein (TSPO) (1:100; Abcam, Cat# ab109497), mouse anti- APC (1:50; Millipore, Cat# OP80), 8-OHG (1:200; QED bioscience, CAT# 12501clone 15A3). The secondary antibodies used were labelled with Alexa 488 or Alexa 555 (Molecular Probes, Leiden, The Netherlands; 1:200), and Hoechst 33342 stain (Sigma, UK; 1 μg/ml PBS) was used to visualize nuclei. Slides were mounted and cover- slipped using Vectashield fluorescent mounting medium (H-1000; Vector Laboratories, Burlingame, CA). For the analysis of NDUFS4 positive cells, we used the EnVision™+ System, HRP (DAB) with rabbit primary antibodies Kit (Dako, CAT #K4011), Rabbit anti- NDUFS4 (1:2000; Abcam, CAT# ab137064). The antibody was diluted using diluent with background reducing components (Dako, CAT #S3022).

For calculation of the lesion size, sections of 7 µm, 200 μm apart and spanning the entire rostro-caudal extent of the injured cortex were stained with haematoxylin and eosin (H&E). The lesion size was measured with ImageJ software (NIH, Bethesda, MD, USA) and calculated using the equation: the contralateral (non-lesioned) hemisphere size minus the injured hemisphere size and divided by the contralateral hemisphere size (Swanson et al, 1990). The results are expressed as a percentage of hemispheric tissue.

### Image capture analysis and processing

Four sections per animal were stained, per antibody. At least 24 fields/section were captured peri-lesionally (immediate area around the lesion). Images were viewed at ×40 and photographed using a Zeiss Axioskop 2 microscope with a Hamamatsu camera (C4742-95). Analyses were carried out by thresholding and then superimposed on nuclei, for co- localization using a dedicated script (JVP AutoColourCellCountsRev) in ImageJ (ImageJ 1.50i, NIH, Bethesda, Maryland). Quantification of microglia morphology was carried out using cell size in ImageJ (minimum 20 cells per animal). The soma perimeter was measured excluding the processes. A Zeiss LSM 710 confocal microscope was used for further characterization (ZENlite software; Zeiss, Cambridge, UK). Figures were prepared using Illustrator software (Adobe Illustrator CS6).

### Brain tissue preparations

Brain tissue from 5 animals per group were used for western blot analysis, phospholipid analysis and the H3 Methylation/Acetylation assay. At day 70 post-TBI, animals were deeply anaesthetized with sodium pentobarbital (50 mg/kg, i.p.; Sagatal, Rhone Merieux, Harlow, UK), and decapitated. Brains, including the cerebellum, were removed and dissected using a brain matrix (as shown in Figure S2C). The contralateral and ipsilateral hemispheres were separated. Tissue was snap frozen and stored at -80 °C.

### Western blot analysis

A cube of the right hemisphere around the lesion was dissected and sonicated in RIPA lysis buffer (Sigma-Aldrich) complete with Protease Inhibitor Cocktail (Sigma-Aldrich), then centrifuged (10,000 x g, 10 min, 4 °C) and the supernatant was taken. Protein concentrations were determined using the Bradford assay. Equal amounts of protein (50 µg) were mixed with NuPAGE® LDS sample buffer (Thermo Fisher Scientific) and dithiothreitol (DTT) and boiled (95 °C, 10 min), then separated using Mini-Protean TGX Gels, 10% (Biorad, UK) and electro-transferred onto polyvinylidene difluoride membranes (Biotrace). Membranes were blocked in 5% non-fat dry milk in Tris-buffered saline (pH 7.4), with 0.1% Tween-20 (Tris-buffered saline-Tween) for 1 h at room temperature. The primary antibodies used were: total The mammalian target of rapamycin (mTOR Rabbit, 1:2,000, Cell Signalling product# 2983, Kit Cat# 9862), p-mTOR (Ser2448; Rabbit 1:2000; Cell Signalling product# 5536, Kit Cat# 9862), phosphorylated p70S6 Kinase (Ser371; Rabbit; 1:1000; Cell Signalling product# 9208, Kit Cat# 9862), all diluted in 5% bovine serum albumin solution, and membranes were incubated overnight at 4 °C. The primary antibody was removed and blots were washed in Tris-buffered saline-Tween and incubated (1 h, room temperature) in horseradish peroxidase-conjugated secondary antibodies (1:10,000; Jackson ImmunoResearch Labs Cat# 323-005- 021). Reactive proteins were visualized using enhanced chemiluminescence (VWR International).

Optical density was determined using ImageJ software (NIH, Bethesda, Maryland). Membranes were also incubated with a mouse vinculin monoclonal antibody (1:4,000; clone VIN-11-5, Sigma-Aldrich Cat# V4505), for normalisation. Protein level was expressed as relative optical density, i.e. the optical density of the band revealed by the primary antibody, divided by the optical density of vinculin in the same lane.

### Phospholipid (PL) analysis

PL analysis was carried out as previously described 22. Lipids were extracted from the cerebellum using the method of Folch and colleagues 23, with 0.01% (w/v) 2,6-di-tert-butyl- p-cresol (butylated hydroxytoluene) as antioxidant. Neutral and acidic PLs were isolated from the lipid extract by solid-phase extraction using Isolute® bonded phase aminopropyl columns (Kinesis Health Technologies, Dublin, UK). The various PL, i.e. phosphatidylcholine (PC), phosphatidylethanolamine (PE), phosphatidylserine (PS), phosphatidylinositol (PI) and sphingomyelin (SM) were separated by thin-layer chromatography, and the phosphate content measured. The results were normalized to 100 mg wet tissue weight.

### H3 Methylation/Acetylation Assay

Histones were extracted from the contralateral hemisphere and were homogenized using the EpiQuik total histone extraction kit (# OP-0006, EpiGentek, Farmingdale, NY, USA) according to manufacturer's directions. EpiQuik™ Global methylation levels of H3K9 (EpiQuik^TM^ Global Histone H3K9 Methylation Assay Kit; CAT# P-3018-96; Epigentek, NY, USA) and global acetyl Histone H3-K9 levels (EpiQuik™ Global Acetyl Histone H3-K9 Quantification Kit CAT# P-4010-96; Epigentek, NY, USA) were determined using a colorimetric ELISA assay as per manufacturer's protocols. Absorbance was measured using a spectrophotometer (450 nm wavelength) with a CLARIOstar® microplate reader and MARS software, and the acetylation and methylation calculated accordingly, with reference to the standard.

### Beta-hydroxybutyrate (β-HB) analysis in plasma

Blood samples were collected (n=10/group) through intracardiac sampling, plasma was prepared by centrifugation (12,000 x g for 5 min) and samples were separated into aliquots. ß- HB was analysed in plasma using the Beta-Hydroxybutyrate Assay Kit (#MAK041; Sigma Aldrich, MO, USA). The assay was performed according to the manufacturer's instructions, with all samples in duplicate. To avoid the presence of interfering substances in the plasma samples, all proteins were filtered out (deproteinized), using a 10 kDa mw cut-off spin filter. A β-HB standard curve was generated in parallel.

### Data analysis

Statistical analysis was performed using GraphPad Prism 8 (GraphPad Software, San Diego, USA). Data was expressed as mean ± sd. Parametric data was analysed using unpaired, two- tailed t-test, one-way ANOVA or two-way ANOVA, following Tukey's multiple comparisons, except when otherwise stated. The level of significance was set to p<0.05. All analysis was carried out blind to experimental condition.

## Results

### KD supplementation improves sensorimotor impairment after CCI

**Figure 1:**
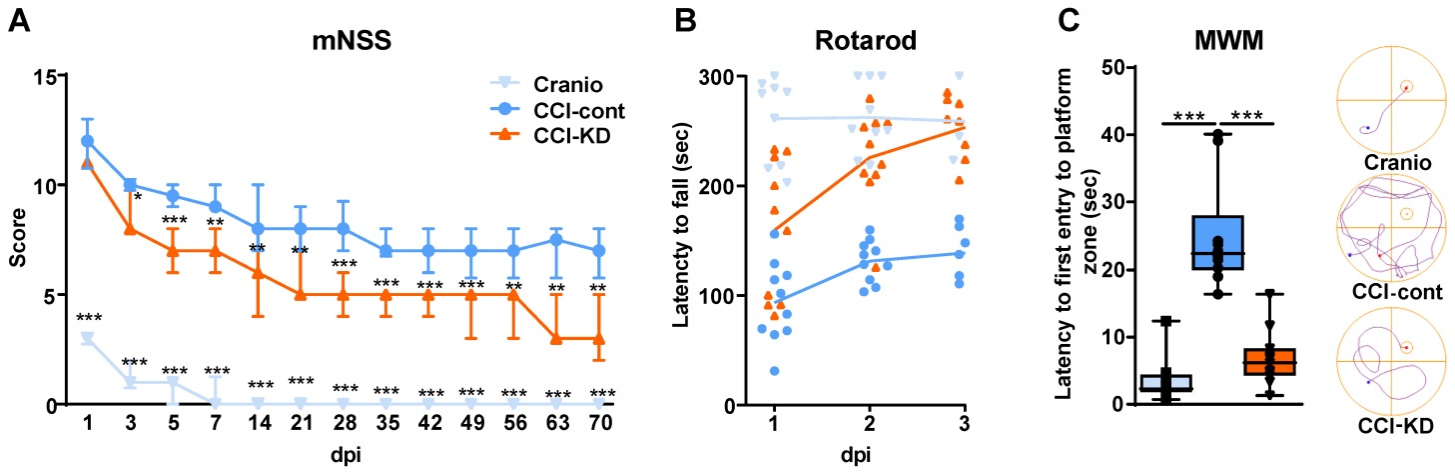
** Motor function assessments.** mNSS (A) and Rotarod (B). (**A**). Line graph showing the dynamic changes in the total scoring in mNSS, starting on the 24 h after the injury and throughout 70 days. From 3 dpi and thereafter, the new-KD fed animals showed a significant improvement compared to the CCI-Control group (Two way ANOVA;***p < 0.0001, F_(2, 28)_ = 247.8 followed by Tukey's multiple comparisons test *p<0.05 (3dpi) **p<0.01 (5dpi) ***p<0.001 (7-70dpi)); the craniotomy group showed significant improvement from 1 dpi and nearly no deficits after 14 dpi (***p<0.001 compared to CCI-Control). (**B**). On 1-3 dpi, both CCI-KD and craniotomy mice showed marked improvement compared with the CCI-Control mice. Graph of individual replicates with connected lines-represents the means. (Two-Way ANOVA; *p*<0.0001, F_(2, 27)_ = 37.46; Tukey's multiple comparisons test: CCI- Control and CCI-KD or craniotomy on dpi 1 (*p*<0.05, *P*<0.001 respectively) and on dpi 2-3 (*P*<0.001). n=10/group. **Cognitive performance assessment with MWM.** (**C**). A significant increase in the latency to the first entry into the platform-quadrant was seen in CCI-Control animals in the probe test compared to the craniotomy group, and this was markedly reduced in the new-KD-treated animals (*One-way* ANOVA; *p* = 0.001, F_(2, 29)_ = 8.262; Tukey's multiple comparisons test ***P<0.001). On the right-hand side, a representative illustration of the track of an animal from each group, from the release point into the water until its first entrance to the platform quadrant. n=10/group.

Following TBI patients suffer from heterogeneous and complex neurological sensorimotor deficits such as lack of muscle coordination, impaired balance, muscle weakness, paresis/paralysis, postural imbalance and gait disturbance. Sensorimotor impairment was assessed with the mNSS and Rotarod tests. TBI led to early significant deficit in the mNSS and all groups showed a gradual decrease in TBI-induced impairment over 70 days. However, a significant improvement was observed in CCI-KD animals already after 3 days post-injury, compared to the CCI-Control group. This difference in mNSS was maintained until the end of the study (Two-way ANOVA; *p* < 0.0001, F (2, 28) = 247.8; Figure 1A). The craniotomy control animals showed only a transient impairment.

The Rotarod revealed better performance in the KD-treated animals compared with the control diet group at all three time points after injury (Two-Way ANOVA; *p* < 0.0001, F (2,27) = 37.46; Figure 1B). The fall latency was significantly higher in the CCI-KD animals, compared to CCI-Control animals. The craniotomy-control group showed minimal impairment in coordination and balance.

### KD improves spatial memory deficits in the MWM after CCI

TBI leads to cognitive deficits in areas such as learning, memory and attention. The hippocampus plays a key role in the acquisition and processing of new memories. A significant hippocampal lesion was apparent in control- CCI animals. The spatial memory was assessed using the MWM. No significant differences were seen between groups during the learning/acquisition phase (Figure S2B). CCI led to significant impairment in the probe trial; the new-KD fed animals showed significantly improved performance in the probe trial compared to the injured animals fed the control diet. (One-Way ANOVA; *p* = 0.001, F (2, 29) = 8.262; Figure 1C).

### KD significantly elevates the plasma levels of ß-hydroxybutyrate after CCI

**Figure 2:**
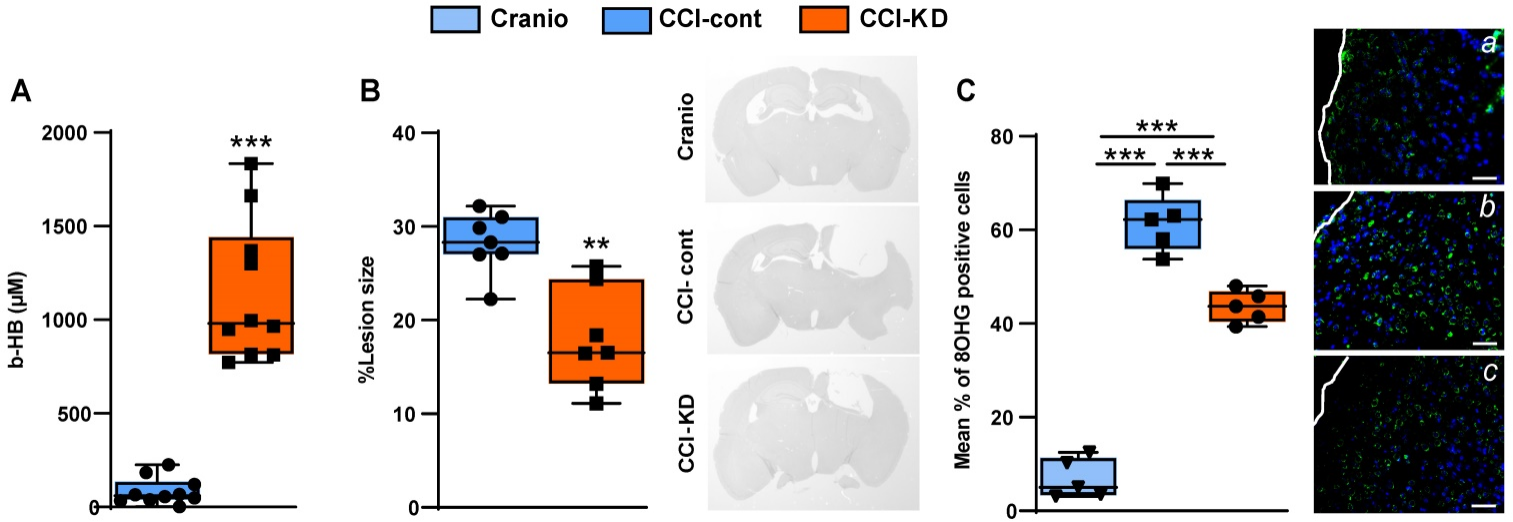
** ß-hydroxybutyrate plasma levels.** (**A**). Higher levels of ß-hydroxybutyrate (ß-HB) were seen 70 days post injury in the new-KD fed animals compared to injured animals fed with control diet; In the craniotomy group ß-HB levels were not detectable (Unpaired *t*-test; ***p < 0.0009, F_(8, 2)_ = 5.709). n=10/group. **Lesion size.** (**B**). Graph showing a significant reduction in lesion size in CCI mice treated with the new-KD compared with CCI-Control vs. craniotomy mice, at 70 days post-TBI (Unpaired *t*-test; *p* =0.01, F_(5, 4)_ = 2.161; **p<0.01) and representative sections stained with H&E showing differences in lesion size. n=5/group. **Oxidative stress.** (**C**). Immunohistochemistry quantification of 8-OHG positive cells around the lesion border 70 days post-TBI (One-way ANOVA *p*<0.0001, F_(3,15)_ = 227.8; Tukey's multiple comparisons test ***p <0.001), and representative images *(a-c)*. n=5/group.

It is well-established that classical KDs are associated with an induction of ketosis, which can be assessed by measuring the β-HB plasma levels. After 70 days of exposure to the diet, a significantly higher plasma β-HB level was detected in the CCI-KD group compared to the CCI-Control animals. Craniotomy controls did not show detectable levels of plasma β-HB in plasma (Unpaired t-test; *p* =0.0009, F (8, 2) = 5.709; Figure 2A).

### KD supplemented animals show a significant reduction in lesion size, a decrease in an oxidation marker and changes in brain PL levels

TBI is associated with rapid loss of grey and white matter that contributes to the overall brain volume loss seen post-traumatically 24. TBI-induced cell loss is associated with necrosis and apoptosis, followed by the removal of cell debris by microglia and macrophages. Analysis of the lesion size after 70 dpi showed a significant loss of tissue, with almost total loss of the ipsilateral hippocampus. The new-KD-fed mice had a significantly decreased lesion size (Unpaired *t*-test; *p* = 0.01, F(5, 4) = 2.161; Figure 2B).

TBI leads to the generation of oxidative stress 25, 26 which we assessed using 8- hydroxyguanosine (8-OHG), a nucleic acid oxidation marker. TBI led to a high level of oxidative damage in the CCI-Control diet group compared to the craniotomy group, and there was a significant reduction in the new-KD-fed animals (One-Way ANOVA; *p* = 0.0001, F (3, 15) = 227.8; Figure 2C; *a-d*).

**Figure 3:**
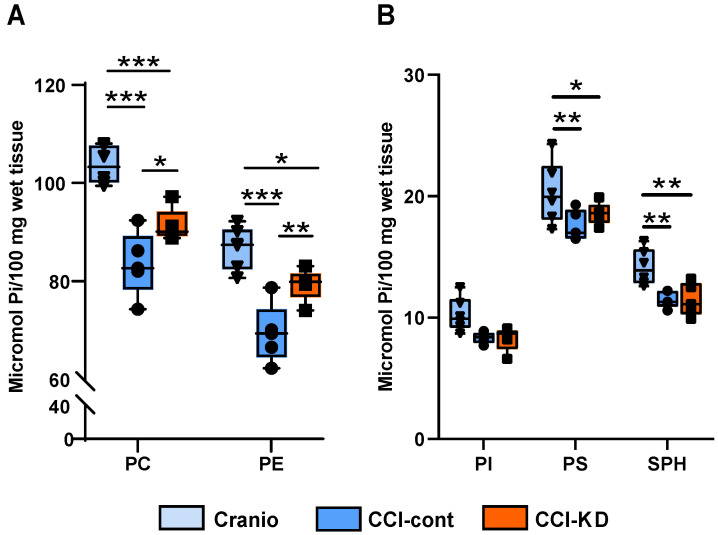
** Lipid analysis.** (**A**). Tissue PC and PE levels were reduced following the brain injury compared to craniotomy controls. This reduction was 12% (PC) or 8.5% (PE) lesser in the new KD group compared with craniotomy controls. Two-way ANOVA; lipid factor: ***P<0.0001 F (1, 26) = 73.73 group factor ***P<0.0001 F (2, 26) = 43.53; Sidak's multiple comparisons test *p<0.05** *p*<0.01*** *p*<0.001). (**B**). Tissue PI, PS and SPH levels were decreased following the injury with no major differences between control and KD diet. Two-way ANOVA; lipid factor: ***P<0.0001, F (2, 39) = 202.2, group factor ***P<0.0001, F (2, 39) = 15.12; Sidak's multiple comparisons test *p<0.05, **p<0.01).

CCI in mice is associated with a decrease in brain PL levels 24. The analysis of the PL in the cerebellum showed that PC and PE levels decreased significantly following the injury compared to the craniotomy group, whereas in the group fed with KD, the loss in PL was reduced. Tissue PC levels were reduced by 20% in injured animals on the control diet vs. craniotomy controls, and this was reduced to 12% in the new-KD group compared with craniotomy controls. PE levels decreased by 20% in the injured animals on the control diet vs. craniotomy-only, while after the new-KD the difference vs. craniotomy controls was only 8.5%. (Two-way ANOVA, *p* < 0.0001; F (2, 26) = 43.53; Figure 3A). Injury also led to decreases in PI, PS and SPH (-19%, -15% and -19% vs. craniotomy-controls, respectively), but the new-KD did not alter these changes (Two-way ANOVA, *P*<0.0001 F (2, 39) = 15.12; Figure 3B).

### KD supplementation leads to a decrease in microglia/macrophage activation but does not alter the astrocyte response post-injury

TBI is followed by intense activation of a microglia and macrophage response. This activation is reflected in morphological changes and is one of the hallmarks of TBI pathophysiology 27. Microglia, the resident brain immune cells, undergo a transition from a resting state- ramified morphology to an amoeboid morphology, which reflects the activated state. In the last decade, it has been shown in human imaging studies that these cells still show signs of activation many years after injury 28. This long-lasting response has also been described in mice and non-human primates 29 and is incompletely understood. We used the classic microglia/macrophage marker, Iba-1, and the translocator protein (TSPO) marker that has been the used extensively for imaging microglia activation state 30. TSPO is expressed in the outer mitochondrial membrane and is present in low concentrations in the healthy brain; it is significantly upregulated in response to brain injury.

**Figure 4:**
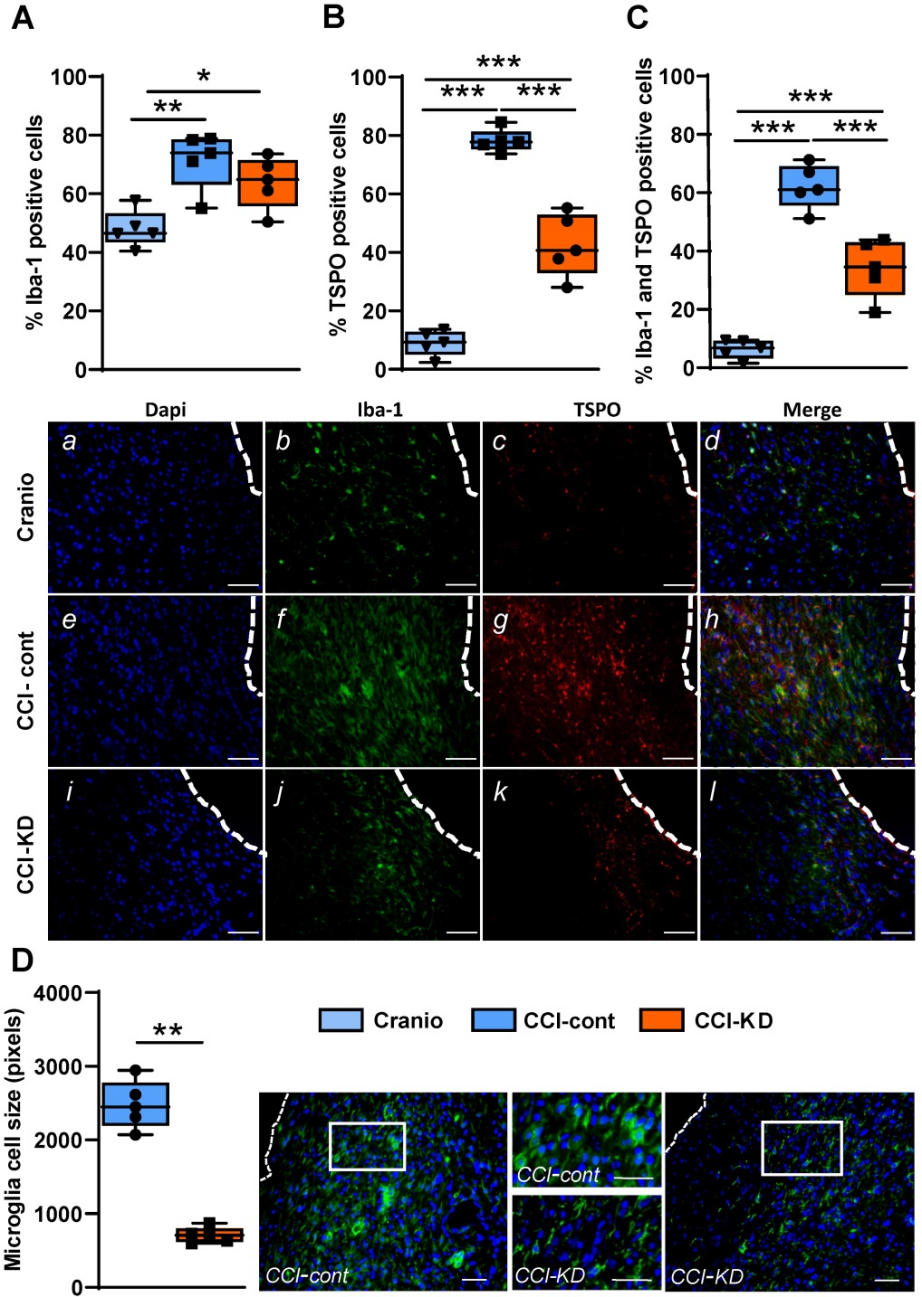
Neuroinflammatory response 70 days post-TBI. Immunohistochemistry quantification around the lesion border, of (**A**) % Iba-1 positive cells (*One-way* ANOVA; *p* = 0.0026, F_(2, 12)_ = 10.15; Tukey's multiple comparisons test *p<0.05 **p<0.01) and representative images (*b,f,j*). (**B**) TSPO positive cells (*One-way* ANOVA; *p* < 0.0001, F_(2, 12)_ = 119.3; Tukey's multiple comparisons test ***p<0.001) and representative images (*c,g,k*). (**C**) Co-localised TSPO and Iba-1 positive cells (*One-way* ANOVA; *p* < 0.0001, F_(2, 12)_ = 68.56; Tukey's multiple comparisons test ***p<0.001) and representative images (*d,h,l*). Note the different microglia morphology (activation): amoeboid vs. ramified. Scale bars=100 µm. To show co-localization we enlarged the area marked with a rectangle. Scale bars= 100 µm. (**D**) Microglia cell size analysis showing the differences in cell size (Mann Whitney test **p<0.01) and corresponding images. The insets show the clear morphological differences. Scale bars= 100 µm. n=5/group.

The percentage of Iba-1 positive cells out of total DAPI, around the lesion was higher in the CCI-Control diet group compared to the CCI-KD and craniotomy groups. (One-Way ANOVA; *p* = 0.002, F (2, 12) = 10.15; Figure 4A, *b, f, j*). Moreover, the new-KD fed mice showed an overall decrease in the percentage of TSPO-positive cells compared to control diet- treated CCI animals (One-Way ANOVA; *p* < 0.0001, F (2, 12) = 119.3; Figure 4B, *c, g, k*).

The co-staining for Iba-1 and TSPO showed a significantly lower percentage of double- stained cells in the CCI-KD group compared to the CCI-Control group (One-Way ANOVA; *p* < 0.0001, F (2, 12) = 68.56; Figure 4C, *d, h, l*). Next, we explored the microglia/macrophage morphological differences between the CCI-Control diet group (predominantly amoeboid), the CCI-KD group (less amoeboid) and craniotomy-control group (predominantly ramified) by measuring cell size. Microglia cells in the CCI-Control diet group were significantly bigger compared with the CCI-KD diet group (Mann Whitney test *p* = 0.008; Figure 4D).

In response to TBI, astrocytes become reactive 31. The astrogliotic reactivity is reflected in both proliferation and cell hypertrophy. After a traumatic injury, astrocytes can exert both harmful and protective effects, e.g. ablation of astrocytes soon after brain injury in mice, led to a poorer outcome, suggesting an important protective role of astrocytes after injury 32, 33.

**Figure 5:**
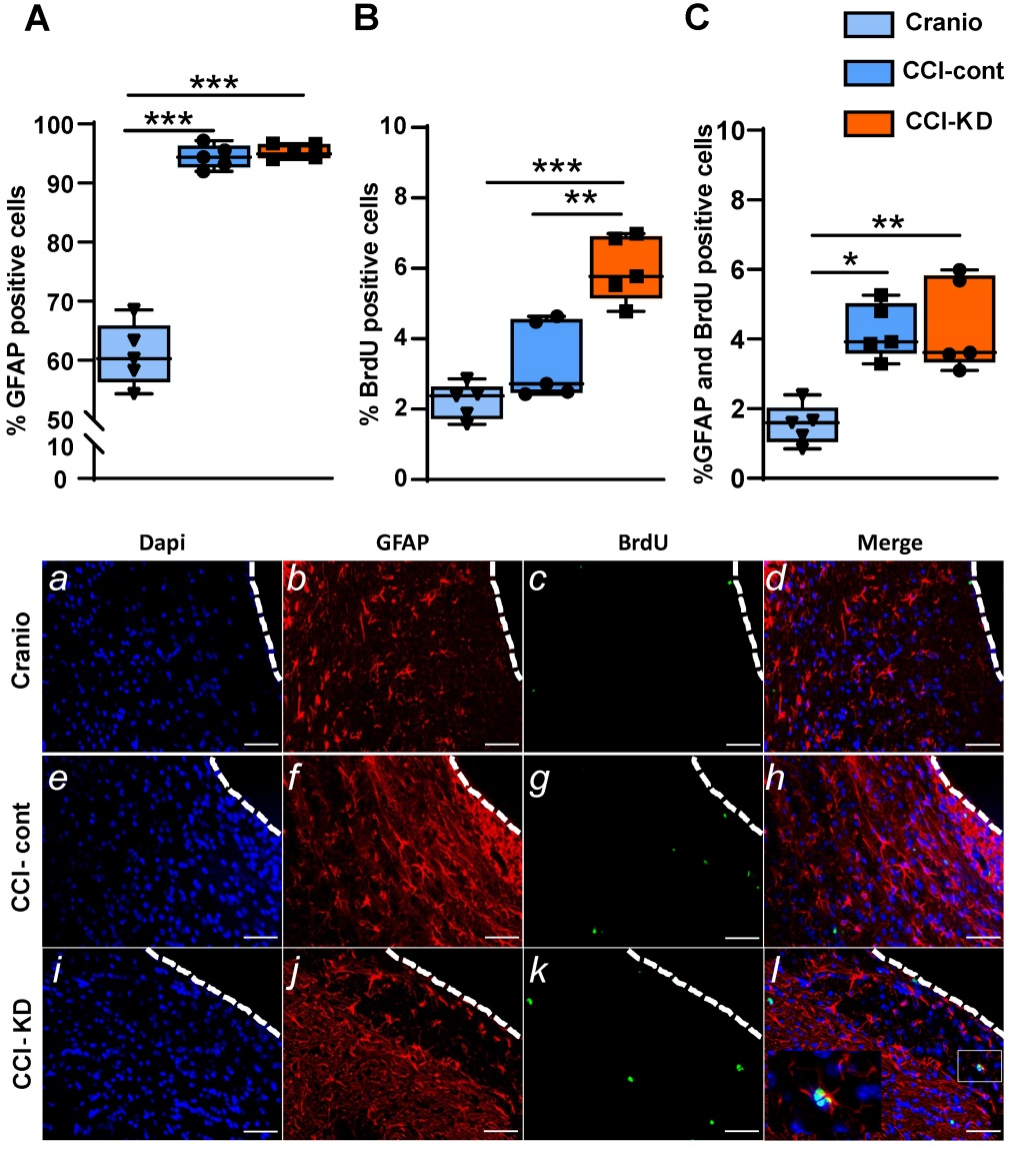
Astrocytic response 70 days post-injury. Immunohistochemistry quantification around the lesion border, of (**A**) % GFAP positive cells (*One-way* ANOVA *p* < 0.0001, F_(2, 10)_ = 118.8; Tukey's multiple comparisons test ***p<0.001) and representative images (*b,f,j*). (**B**). % BrdU positive cells (*One-way* ANOVA *p* < 0.0001, F_(2, 12)_ = 23.71; Tukey's multiple comparisons test **p<0.01***p<0.001) and representative images (*c,g,k*). (**C**). Co-localised % GFAP and BrdU positive cells (*One-way* ANOVA; *p* < 0.0001, F_(2, 10)_ = 9.652; Tukey's multiple comparisons test *p<0.05 **p<0.01) and representative images (*d,h,l*). n=5/group.

CCI led to an increase in the percentage of the positive astrocyte marker- glial fibrillary acidic protein (GFAP), out of total DAPI - around the lesion border, with no differences between the control diet and the new-KD diet groups (One-Way ANOVA; *p* < 0.0001, F (2, 10) = 118.8; Figure 5A, b, f, j). Additionally, in both diets, after TBI, there was an increase in newly- formed astrocytes, i.e. cells double-labelled with GFAP and BrdU, at 70 dpi (One-Way ANOVA; *p* = 0.005, F (2, 10) = 9.652; Figure 5C, *d, h, l*).

### KD supplementation modulates cell proliferation after CCI

TBI is associated with an increase in cell proliferation, including an induction of neurogenesis 34 35, processes which are interpreted as repair mechanisms. New-born cells are detected not only in the neurogenic areas of the brain (such as the subgranular zone of the dentate gyrus or the subventricular zone of the lateral ventricles), but also in the perilesional area, and in contralateral hemisphere regions such as the hippocampus. The analysis of proliferating cells after CCI around the lesion, showed a significantly higher percentage of BrdU-positive cells compared to the craniotomy group. The treatment with KD led to a noticeably increased perilesional cell proliferation (One-way ANOVA; *p* < 0.0001, F (2, 12) = 23.71; Figure 5B, *c, g, k*).

### KD supplementation leads to the protection of oligodendrocytes after CCI

**Figure 6:**
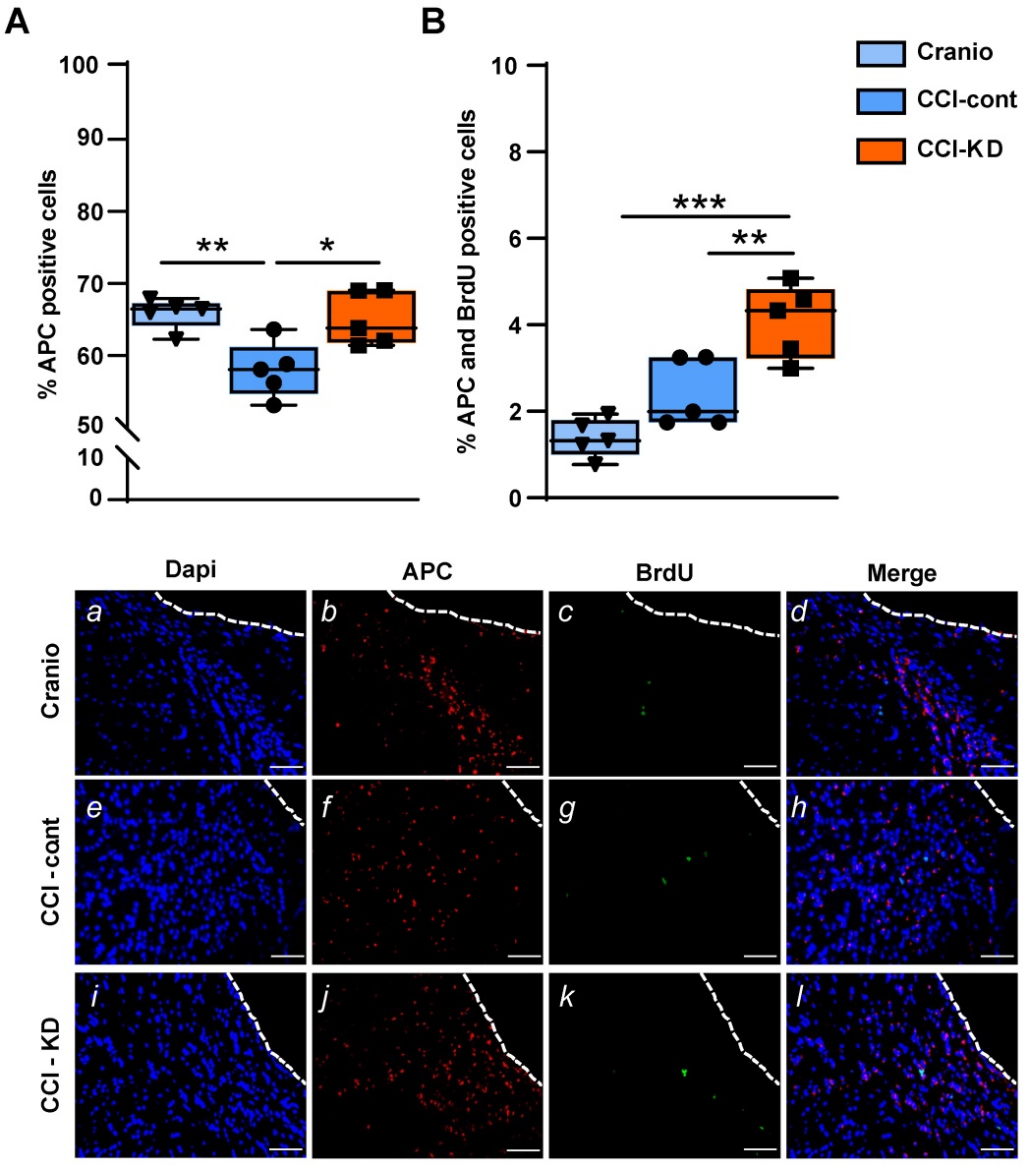
** Oligodendrocytes.** (**A**). Quantification of oligodendrocytes 70 days post-TBI (APC positive cells) (*One-way* ANOVA *p* = 0.005, F_(2, 12)_ = 8.563; Tukey's multiple comparisons test **p<0.01 ***p<0.001) and representative images (*b,f,j*)). (**B**). Dual staining APC and BrdU (*One-way* ANOVA *p* = 0.0002, F_(2, 12)_ = 18.15; Tukey's multiple comparisons test *p<0.05 **p<0.01) and representative images (*d,h.l*). Scale bar=100 µm. n=5/group.

Oligodendrocytes are responsible for producing myelin and maintaining myelinated fibres in the brain. TBI alters the response of both mature oligodendrocytes and immature proliferative oligodendrocytes 36. We show that the CCI injury led to a significant reduction in the percentage of oligodendrocytes, labelled with adenomatous polyposis coli (APC) out of total DAPI, perilesionally. This injury-induced decrease was significantly affected by the new-KD (One-Way ANOVA; *p* = 0.005, F (2, 12) = 8.563; Figure 6A, b, f, j). In addition, dual staining of APC and BrdU showed a higher percentage of double-stained APC/BrdU cells in the CCI-KD group compared with the two other groups (CCI-Control 2.4±0.35; CCI-KD 4.1±0.38; Craniotomy 1.4±0.2; One-way ANOVA; *p* = 0.002, F (2, 12) = 18.15; Figure 6B, *d, h, l*).

### KD modulates mTOR signalling and alters the mitochondrial marker NDUFS4 levels after CCI

**Figure 7:**
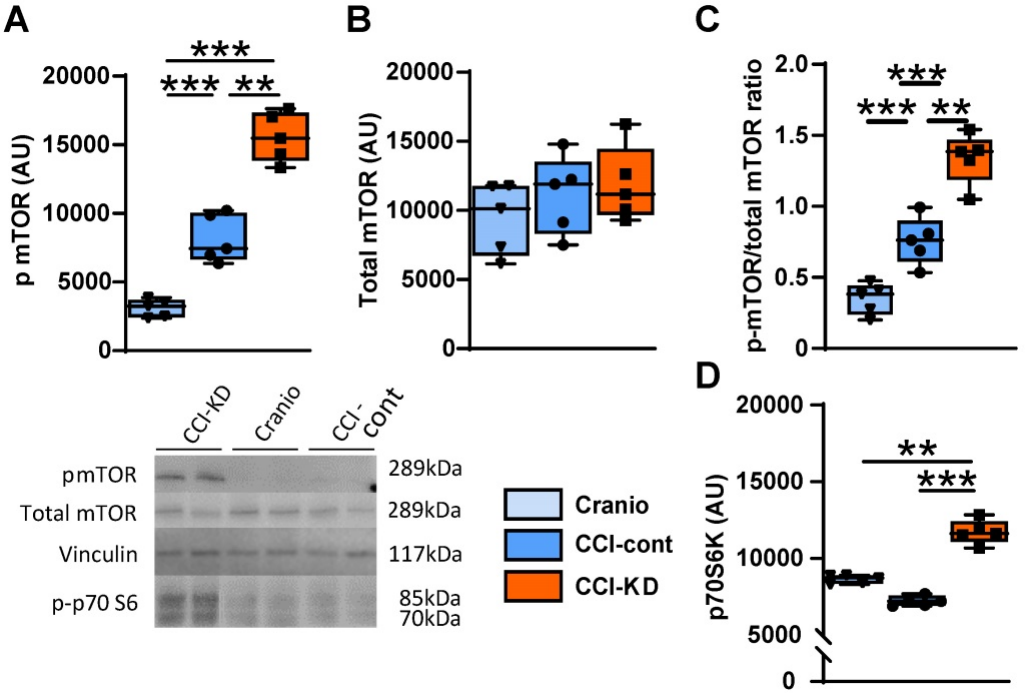
** mTOR pathway.** Graph and representative cropped gels of western blot analysis of protein levels of: (**A**). Phosphorylated mTOR (Ser2448) (289 kDa) (One-way ANOVA *p* < 0.0001, F_(2, 11)_ = 37.44; Tukey's multiple comparisons test **p<0.01, ***p<0.001), (**B**). Total mTOR; 289 kDa (One-way ANOVA: ns, F_(2, 14)_ = 0.5253; Tukey's multiple comparisons test: ns), and (**C**). Phosphorylated-mTOR/total mTOR ratio.

The mammalian target of rapamycin (mTOR) is a protein kinase that integrates energy, nutrient and growth factor signals to regulate various cellular functions, when activated. It is part of two distinct multi-protein complexes, mTORC1 and mTORC2. The activation of mTOR has been associated with repair mechanisms after neural injury 37, 38. One of the downstream signalling effectors in the mTORC1 pathway is p70S6k (ribosomal protein S6 kinase beta-1). There is an increase in the levels of phosphorylated mTOR (p-mTOR) in the injured tissue and the new-KD further amplified this response to injury. Thus, the ratio of p-mTOR/mTOR, at 70 days after injury, significantly increased in the CCI-KD group compared to both CCI-control and craniotomy groups (1.34±0.08 vs. 0.75±0.07 and 0.34±0.05 respectively; One-Way ANOVA ***p<0.0001, F (2,12) = 51.26; Figure 7C). The new-KD also increased the level of the mTORC1 downstream effector p70S6k in its phosphorylated form at 70 days post injury compared to the CCI-Control and craniotomy groups (p-mTOR: One-Way ANOVA; *p* = 0.001, F (2, 11) = 37.44; Figure 7A, B and p70S6k: One-Way ANOVA; *p* = 0.001, F (2, 7) = 44.72; Figure 7D).

**Figure 8:**
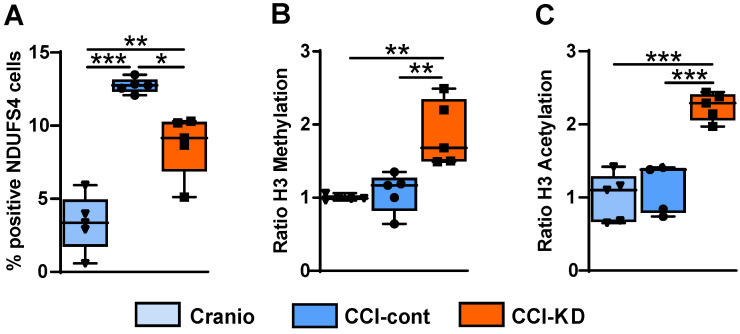
** Complex-I mitochondrial marker.** (**A**). Quantification of NDUFS4 positive cells 70 days post-TBI (One-way ANOVA *p* = 0.0006, F_(2, 9)_ = 19.18; Tukey's multiple comparisons test *p<0.05, **p<0.01 ***P<0.001). **Epigenetics markers.** Quantification of (**B**). H3K9 Methylation ratio (One-way ANOVA *p* =0.0004, F_(3, 15)_ = 11.33; Tukey's multiple comparisons test **p<0.002) and (**C**). H3K9 Acetylation ratio (One-way ANOVA *p* <0.0001, F_(3, 15)_ = 20.71; Tukey's multiple comparisons test ***p <0.001). All measurements were assessed at 70 days post-TBI. n=5/group.

TBI induces acutely a significant mitochondrial dysfunction 39. The protein NADH dehydrogenase [ubiquinone] iron-sulfur protein 4 (NDUFS4) is an accessory subunit of the mitochondrial membrane respiratory chain NADH dehydrogenase (complex I). It is a membrane protein located in the inner mitochondrial membrane and it is part of the mitochondrial respiratory chain that plays a vital role in cellular ATP production. At 70 days post-injury we detected a marked increase in NDUFS4, and the levels of this mitochondrial marker NDUFS4 in the perilesional area were reduced in the injured mice receiving the new- KD (One-Way ANOVA; *p* = 0.0006, F (2, 9) = 19.18; Figure 8A).

### KD modulates H3K9 methylation and acetylation

In the last decade, evidence has been accumulating that ketones can induce epigenetic modifications, such as increased histone acetylation and DNA methylation 40. A strong effect of the new-KD was observed on both histone H3K9 methylation and acetylation compared with the CCI-Control and craniotomy groups (One-Way ANOVA; *p* = 0.0004, F (3, 15) = 11.33; Figure 8B and One-Way ANOVA; *p* < 0.0001, F (3, 15) = 20.71; Figure 8C respectively).

## Discussion and Conclusion

In this study, we carried out a TBI in adult mice using the CCI model - which was previously shown to be insensitive in adult animals to ketogenic dietary intervention 13 and fed the animals a new-KD for 70 days post-injury, while assessing the neurological outcome. The CCI model involved replacing of the bone flap and allowing expansion, therefore the observations presented with this new diet can be interpreted as reflecting a clinical situation where there is control of the acute rise in intracranial pressure following a severe injury. We also examined the brain tissue at the end of the experiment, for signs of neuroprotection. We present evidence that a new ketogenic formulation given immediately post TBI in adult animals can lead to functional neurological benefits and can reduce the injury impact on brain tissue. This new preparation has a lower fat to carbohydrate and protein ratio, i.e. a ratio of 2:1 vs. a ratio of 4:1, which is characteristic of classical KD formulations, and includes medium chain triglycerides (MCT), docosahexaenoic acid (DHA) and leucine. It is also higher in protein, fibres and carbohydrates with low glycaemic index, compared with the classic KD.

Intervention with this novel KD formulation reduced sensorimotor impairment, including coordination and balance, and improved spatial memory recovery after injury. Tissue loss post-injury is linked to the sensorimotor and cognitive deficits seen after injury 41. The brain lesion size was significantly decreased in the new-KD group compared to the injured animals on control diet, and the PL loss in tissue following injury was also attenuated.

TBI initiates a series of neurochemical events that compromise cerebral energy metabolism. This metabolic disruption requires high cellular energy to reinstate homeostasis. TBI leads to a transient “hyperglycolysis” phase (possibly associated with the acute post-injury increase in hypoxia-inducible factor 1-alpha 42), followed by a prolonged period of metabolic depression, during which glucose utilisation drops dramatically. Although glucose serves as the main metabolic substrate for neurons, it has been shown that other energy-producing substrates, such as lactate and pyruvate, might be utilized by neurons to sustain their activity 15. In addition to glucose metabolism, all cell types in the brain are capable to metabolise ketone bodies when present, and ketones are capable of fulfilling the energy requirements of the brain. Ketosis, reflected in elevated β-HB plasma levels, can be achieved by following a high-fat, low-carbohydrate classical KD that acts as a provider of alternative substrates for cerebral energy metabolism 6. We show here that the β-HB plasma levels were significantly higher in the injured group fed with the new-KD, emphasising the fact that this formulation can lead to a state of ketosis, although the ketogenic ratio is lower than in the classical KD. The β-HB level we measured is in the range reported in previous studies with KD formulations in rats and mice 43-45.

The new-KD modified the neuroinflammation reaction post-injury, i.e. it reduced activated microglia peri-lesionally. Numerous studies have reported that post-injury changes in the microglia functional state are associated with the *de novo* expression of the mitochondrial translocator protein (TSPO). Post-TBI, there is an increase in TSPO levels 46, 47. Here we show that injured animals fed with the new-KD had a significantly reduced TSPO expression in Iba-1 expressing cells compared with the injured animals fed with a control diet, at 70 days post-CCI.

One other component of the glial response after TBI is represented by astrocytic activation. Astrocytes respond to mechanical strain and to the ATP released from injured cells at the injury site, which triggers astrocyte activation and recruits them to the site of injury. In response to a focal injury, reactive astrocytes form a scar border that isolates the damaged and inflamed tissue from the healthy tissue 48-50. These scar borders are comprised almost entirely of newly proliferated astrocytes, which may subsequently contribute to tissue repair/regeneration 33, 51. The present study indicates a strong astrocytic activation at more than 2 months after injury, as reflected by the GFAP marker, and also shows an increase in the numbers of newly-born astrocytes even at this late time post-injury, but with no differences between the control diet and the new-KD treated groups.

TBI triggers loss of oligodendrocytes and demyelination, and there is a relationship between white matter loss and post-traumatic cognitive impairment 52, 53. The current findings confirm the oligodendrocyte vulnerability after traumatic injury and indicate a protection of oligodendrocytes and also an increase in the newly formed oligodendrocyte pool along the lesion border, by the new-KD. As previously shown, CCI leads to a detectable decrease in PL species at this time point after injury 54. The new-KD reduced the decrease in PC and PE, but, overall, the impact on PL loss in tissue was not as marked as the impact of a dietary supplementation with a combination of brain phospholipid precursors 54.

Studies on KD have shown that these fat-rich and carbohydrate-poor diets can induce epigenetic alterations, and it is interesting to note that epigenetic changes such as increased histone acetylation have been associated with improved learning and memory, while decreased acetylation is associated with cognitive impairment such as that seen during ageing 55, 56. Gao and colleagues 57 have reported decreases in histone acetylation and methylation after TBI in the immature brain. Our new-KD induced a marked increase in histone acetylation and in histone methylation, therefore it could be suggested that this epigenetic modulatory effect might be beneficial post-TBI.

Mitochondrial dysfunction is an early event post-trauma, and is a critical determinant of energy failure and cellular demise 58. Mitochondria disruption is also a key link to increased oxidative stress 59, which appears to be reduced by the new-KD in the injured CCI tissue.

Interestingly, our results show that injury leads to an increase in the tissue expression of the mitochondrial marker NDUFS4, and the new-KD significantly dampened this increase. The knockout of NDUFS4 in the heart has been shown to be protective in reperfusion injury following heart ischaemia 60. Furthermore, it has been shown that the activation state of mitochondrial complex I is linked to reperfusion injury and oxidative stress post-ischaemia 61. An improved understanding of the impact of the new-KD on brain mitochondria at this time after injury and a comprehensive interpretation of these first observations on the complex I marker, would be greatly aided by functional measures on fresh mitochondria isolated from the injured tissue, and a characterization of the transitions between conformational states (active vs. de-active and catalytically dormant) of complex I, as reported in recent studies in a murine model of immature hypoxia-ischaemia encephalopathy 62.

One of the changes reported in studies with KD in the context of epilepsy is an inhibition of mTOR 63, a signalling pathway whose activation is linked to epileptogenesis. It has been suggested that this inhibitory effect may be linked to the impact of KD on growth and also its anticonvulsant properties. It has been shown that TBI leads to a fast activation of mTORC1, and suppression of this initial activation may be beneficial 64. We show that CCI in mice led in the long-term to an activation of mTOR (as reflected in the increase in p-mTOR), and this was enhanced by the new-KD, after 70 days of exposure to this diet. This enhancement in signalling was supported by the observed increase in phosphorylated p70S6k. It is tempting to suggest that this effect, which is reported for the first time after traumatic central nervous system injury, may be linked to beneficial effects of mTOR activation on compensatory brain plasticity post-injury and recovery of cognitive function 65, 66; effects which are thus clearly opposite to the deleterious impact of mTOR activation in the acute phase. Although the new protein synthesis which would be involved in endogenous attempts to restore brain connectivity may not be exclusively under the control of mTOR (in particular mTORC1), this signalling pathway may still make a significant contribution to the reduction of the lesion and the inherent brain circuit remodelling involved in improved neurological performance.

Interestingly, an increase in mTOR activation has also been reported after chronic exposure to a KD treatment which induced neuroprotection in a model of optic neuropathy 67. In the present study, it is likely that the addition of leucine to the new-KD may have contributed to this effect. It would be interesting to assess in future studies whether the early post-injury effect of the new diet is to inhibit mTOR, whereas in time, the effect becomes opposite, and leads to stimulation of mTOR, which supports neurorepair.

Overall, we show evidence in the present study that it is possible to characterize significant neuroprotective properties of a KD following TBI in an adult brain, using a new-KD formulation with a fat to carbohydrate and protein ratio which is also likely to be associated with an improved tolerability, and also enriched in omega-3 fatty acids, in particular, DHA, which has been shown to have intrinsic neuroprotective value in TBI. With reference to the DHA diet content of our new diet (1430 mg/100 g diet), it is interesting to note that Pu and collaborators 68 administered for 35 days a diet enriched in fish oil (4000 mg fish oil/100 g diet, containing DHA and eicosapentaenoic acid (EPA); EPA:DHA ratio of 2.6) following CCI in mice, and reported improvement in tissue protection and in functional outcome after injury. As our first exploratory study was focused on the proof of principle for the whole diet, it is not possible to establish which diet component made the most critical contribution to the benefits seen on various endpoints, and this will need to be addressed with priority in future studies, to refine further the composition of the diet and better understand mechanistically its mode of action. Future studies could help assess whether the beneficial long-term effects of the new diet are supported by very early neuroprotective mechanisms triggered in the first hours or day post-injury by the diet. This is the first evidence which suggests that a KD approach could be used in human adult TBI, expanding the range of potential clinical uses of this dietary KD principle 69 in an indication where there is a need to move away from glucose as a fuel and also avoid hyperglycaemia 70, which is a modifiable risk factor contributing to poor outcome in TBI 71. How this dietary paradigm could be implemented post-trauma in the context of human TBI, and the exact dietary regime parameters, i.e. optimum onset and duration of the intervention, remain to be clarified in further studies.

## Supplementary Material

Supplementary figures and tables.

## Author's contributions

OTZ, AMT and MV designed the experiments. OTZ carried out *in vivo* and *ex vivo* work and analysed the data. JLT and OTZ carried out the surgery. RJH, NvW and LMB designed the diet. LS; RGF and BK contributed to immunohistochemistry analysis. SCD carried out lipid analysis. MR and UPE carried out the epigenetic analysis. JVP wrote the script for ImageJ software. SL measured β-HB levels. OTZ and AMT wrote the manuscript. MV, JPS, JLT, RJH, LMB, BK, RGF and NvW reviewed the manuscript.

## Figures and Tables

**Table 1:** Composition of the experimental diets; control (cont.) vs. the new ketogenic diet (KD) (g/100 g)

Diet compositions	Control	New KD
	g / 100g diet	g / 100g diet
**Fats**		
Soy oil	1.90	
Corn oil	2.20	
Coconut oil	0.90	4.40
Tuna oil		5.70
Palm oil		39.90
Rapeseed oil		5.70
MCT oil		1.30
**Proteins**		
Caseine (85%)	14.00	18.70
L-Cystine	0.18	0.30
L-Leucine		1.90
**Carbohydrates**		
Cornstarch, pre-gelatinized	45.59	
Maltodextrin, 10 DE	15.50	
Sucrose	10.00	
Galactose		1.50
Isomaltulose		2.72
**Fibres**		
Cellulose powder	5.00	5.00
Fructooligosaccharides		5.00
**Vits, Mins, Additions**		
Mineral & trace element premix (AIN-93M-MX)	3.50	5.83
* [including 22% Sucrose]*	*0.77*	*1.28*
Vitamin mix (AIN-93-VX)	1.00	1.67
* [Including 97% Sucrose]*	*0.97*	*1.62*
Choline chloride (50%; 0,434 g / g)	0.23	0.38
Tert-butylhydroquinone	0.0008	0.0013
	***Control***	***New KD***
	***mg / 100g diet***	***mg / 100g diet***
**Fatty acid compositions**		
C-8:0	64.4	897.3
C-10:0	51.5	699.1
C-12:0	395.0	2066.6
C-14:0	154.6	1317.0
C-16:0	492.4	18064.5
C-18:0	136.1	2285.2
C-18:1w9 (OA)	1040.6	19313.1
C-18:2w6 (LA)	2180.8	5055.2
C-18:3w3 (ALA)	107.4	527.6
C-20:5w3 (EPA)	0.0	326.3
C-22:6w3 (DHA)	0.0	1430.4
*A. Total Fats*	*5.0*	*57.0*
*B. Total Proteins*	*12.1*	*18.1*
*C. Total Carbohydratess*	*71.1*	*4.2*
*D. Total Sucrose from Vits & Mins*	*1.7*	*2.9*
*Ketogenic ratio [A/(B+C+D)]*	*1 : 17*	*2.3 : 1*
**Energy (kcal/100g diet)**	**377**	**626**

## Abbreviations

ATP: adenosine triphosphate
β-HB: beta-hydroxybutyrate
BrdU: 5-bromo-2-deoxyuridine
CCI: controlled cortical impact
DAPI: 4′,6-diamidino-2-phenylindole
DHA: docosahexaenoic acid
dpi: days post-injury
DTT: dithiothreitol
GFAP: glial fibrillary acidic protein
IHC: immunohistochemistry
KB: ketone bodies
KD: ketogenic diet
MCT: medium chain triglycerides
mNSS: Modified Neurological Severity Score
mTOR: mammalian target of rapamycin
MWM: Morris water maze test
NDUFS4: NADH dehydrogenase [ubiquinone] iron-sulfur protein 4 (NDUFS4)
8-OHG: 8-hydroxyguanosine
PBS: phosphate-buffered saline
PC: phosphatidylcholine
PE: phosphatidylethanolamine
PFA: paraformaldehyde
PI: phosphatidylinositol
PL: phospholipid
PS: phosphatidylserine
SM: sphingomyelin
TBI: traumatic brain injury
TSPO: translocator protein
