# Supplementary material for: A new ketogenic formulation improves functional outcome and reduces tissue loss following traumatic brain injury in adult mice
Source: Theranostics. 2021 Jan 1;11(1):346–60. doi: 10.7150/thno.48995 (PMC7681084; doi:10.7150/thno.48995)
Supplement: Supplementary file 1 — Supplementary figures and tables. [file thnov11p0346s1.pdf]

			Macronutrients %en							
Type	Identifier	KD ratio	Fat	Protein	Carb	kcal/100g	Fibres	n-3 PUFAs	MCTs	Reference
Control	Teklad #7013	1:10	18	23	59	308	4%	0.30%	0%	Appelberg 2009
	AIN93M	1:12	16	18	66	391	5%	0.48%	0%	Ma 2018
	Purina 5001	1:15	14	29	58	334	5%	0.19%	--	Elamin 2017
	BioServ F5960	1:17	12	19	69	379	--	0.04%	--	Steijger 2013
	**Control**	**1:17**	**12**	**13**	**76**	**377**	**5%**	**0.11%**	**0.5%**	**current study**
KD	BioServ F3666	6:1	93	5	2	724	5%	0.67%	--	Appelberg 2009; Elamin 2017
	TP-201450	6:1	93	5	2	734	--	--	--	Ma 2018
	BioServ F5848	3:1	88	11	2	677	--	0.56%	--	Steijger 2013
	**New KD**	**2:1**	**84**	**12**	**5**	**626**	**10%[a]**	**2,28%[b]**	**3.7%**	**current study**

Table S1: Overview of the current Control diet and New KD diet characteristics as compared to some of the previously reported Control and KD diets. KD ratio = (fat w/w) : ((protein w/w) + (carb w/w)). Carb = carbohydrates; MCTs = medium chain triglycerides; -- = unknown. [a] incl. 5% FOS. [b] incl. 1,76% DHA+EPA. Note that the current New KD has a low KD ratio, caused by a relatively low fat percentage and a relatively high carb percentage. It also has a high fibre content, with an additional 5% fructooligosaccharides (FOS) next to the usual 5% of cellulose. The New KD contains the omega-3 polyunsaturated fatty acids (n-3 PUFAs) docosahexaenoic acid (DHA) and eicosapentaenoic acid (EPA). Finally, the New KD contains relatively high amounts of MCTs.

Appelberg KS, Hovda DA, Prins ML. The effects of a ketogenic diet on behavioral outcome after controlled cortical impact injury in the juvenile and adult rat. J Neurotrauma. 2009; 26: 497-506.

Elamin M, Ruskin DN, Masino SA, Sacchetti P. Ketone-Based Metabolic Therapy: Is Increased NAD(+) a Primary Mechanism? Front Mol Neurosci. 2017; 10: 377.

Ma S, Huang Q, Tominaga T, Liu C, Suzuki K. An 8-Week Ketogenic Diet Alternated Interleukin-6, Ketolytic and Lipolytic Gene Expression, and Enhanced Exercise Capacity in Mice. Nutrients. 2018; 10.

Streijger F, Plunet WT, Lee JH, Liu J, Lam CK, Park S, et al. Ketogenic diet improves forelimb motor function after spinal cord injury in rodents. PLoS One. 2013; 8: e78765.

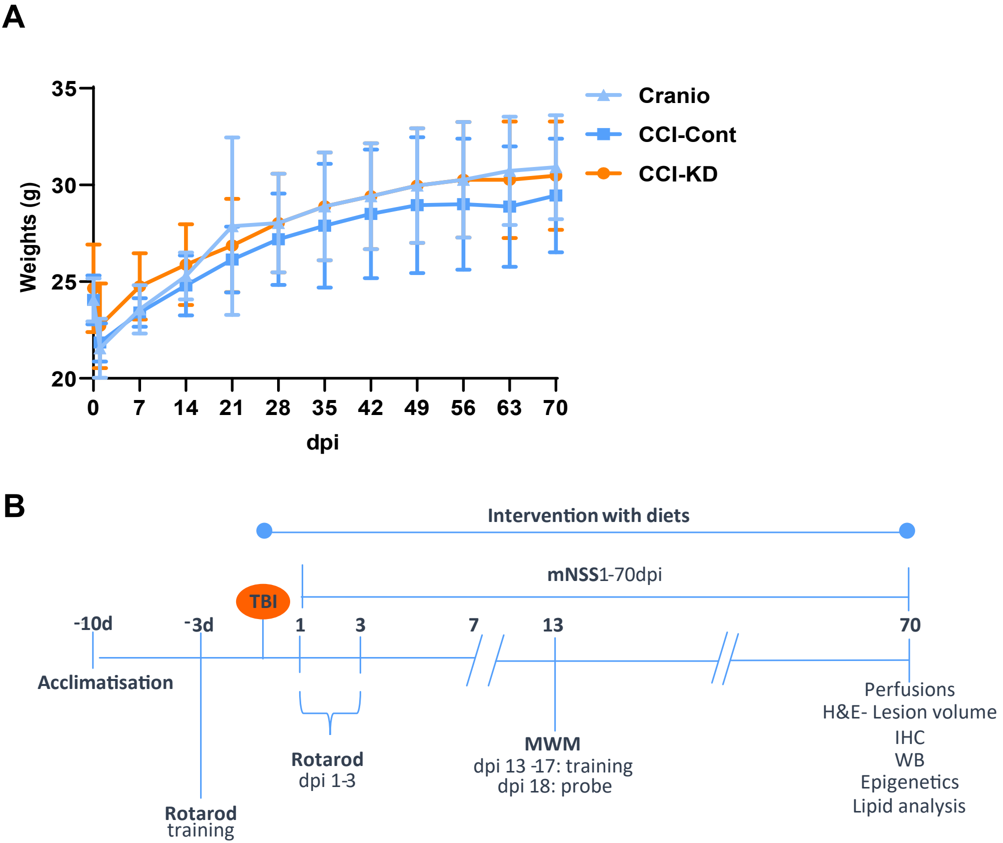

* dpi refers to days post injury

* Daily monitoring of weights and food

Figure S1. Body weights comparison and experimental design.
A. Graphs of weekly measurements of group-weights average showing no differences between groups.
B. Experimental design. The behavioural testing was performed for 70 days post-injury (dpi). A controlled cortical impact (CCI) or a control craniotomy (sham injury) were induced in adult male mice. Animals were divided into 3 experimental groups (craniotomy-control, CCI-Control and CCI-KD). All mice were tested for motor and cognitive impairments. Mice were trained for three consecutive days for the Rotarod test prior to injury. Throughout the study (1-70 dpi), mice were tested for mNSS every other day on the first week and once a week thereafter, Rotarod (1-3 dpi) and MWM (13-18 dpi). A week before the end of the study mice were injected twice a day with BrdU for the remaining time. Body weight were monitored daily. On 70 dpi mice underwent perfusion for immunohistochemistry (IHC) analysis or were decapitated and brains were quickly removed and snap frozen for western blot (WB) analysis, lipid analysis and epigenetic marker measurements. 70 dpi plasma samples were collected and used for β-Hb quantification.

A

Task	Score
*Exit- time**	1-3
Walking straight	1
Startle reflex	1
Seeking behaviour	1
Hemiparesis	1
Round stick balance	1
Triangle stick balance	1
*Beam walking***	
1cm	1-3
2cm	1-3
3cm	1-3
Total score	10 - 18

**Exit task- points*	
exit within 20 sec	0
exit within 60 sec	1
exit within 2 min	2
no exit within 2 min	3
***Beam walk-points*	
Balance and walk with normal posture	0
Grasp side of the beam+ draging OR sliping poterior paw less than 3	1
More than 3 times and no abiliti to grip	2
Unable to walk the beam	3

B

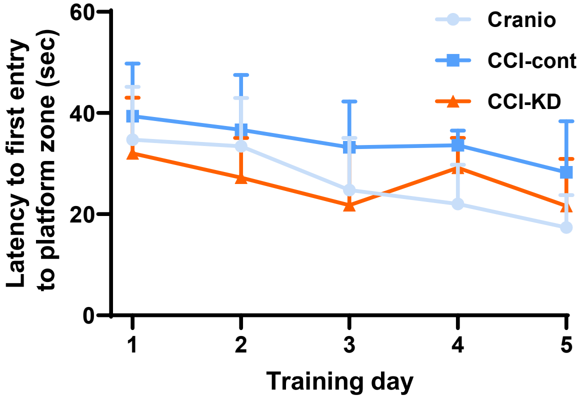

C

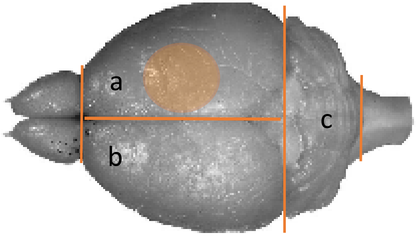

Figure S2. Modified neurological severity score test (mNSS), MWM-learning curve and Brain tissue process illustration.

A. Content of the Modified Neurological severity scores (NSS) outlined in Table. This modified test consists of 10 individual clinical parameters, including tasks on motor function, alertness and other neurophysiological indicators, to evaluate the neurological impairment. One point is awarded for the inability to perform the tasks. A maximal NSS of 18 points thus indicates severe neurological dysfunction, with failure on all tasks. Modifications of the scoring are represented with asterisks and detailed respectively in the second table.

B. Morris water maze acquisition performance graph. Over the course of five consecutive days, mice were given a series of four daily trials. Data are average of the latency (sec) to reach the goal (platform), using identical sequences of start locations.

C. Dorsal view of a mouse brain illustrating the three sections that has been processed; a. Ipsilateral hemisphere (right side), containing the injury/lesion, used for western blot analysis. b. Contralateral hemisphere (left side), used for H3 Methylation/Acetylation Assay and c. Cerebellum, used for phospholipid analysis.
